# Magnolol promotes the autophagy of esophageal carcinoma cells by upregulating *HACE1* gene expression: Magnolol activates HACE1-mediated autophagy

**DOI:** 10.3724/abbs.2024044

**Published:** 2024-04-25

**Authors:** Kenan Huang, Biao Zhang, Yu Feng, Haitao Ma

**Affiliations:** 1 Department of Thoracic Surgery Dushu Lake Hospital Affiliated to Soochow University Suzhou 215000 China; 2 Department of Thoracic Surgery Shanghai Changzheng Hospital Navy Military Medical University Shanghai 200003 China

**Keywords:** magnolol, esophagus cancer, autophagy, HACE1, OPTN

## Abstract

Esophagus cancer (EC) is one of the most aggressive malignant digestive system tumors and has a high clinical incidence worldwide. Magnolol, a natural compound, has anticancer effects on many cancers, including esophageal carcinoma, but the underlying mechanism has not been fully elucidated. Here, we first find that magnolol inhibits the proliferation of esophageal carcinoma cells and enhances their autophagy activity in a dose- and time-dependent manner. This study demonstrates that magnolol increases the protein levels of LC3 II, accompanied by increased HACE1 protein levels in both esophageal carcinoma cells and xenograft tumors. *HACE1*-knockout (KO) cell lines are generated, and the ablation of *HACE1* eliminates the anti-proliferative and autophagy-inducing effects of magnolol on esophageal carcinoma cells. Additionally, our results show that magnolol primarily promotes *HACE1* expression at the transcriptional level. Therefore, this study shows that magnolol primarily exerts its antitumor effect by activating HACE1-OPTN axis-mediated autophagy. It can be considered a promising therapeutic drug for esophageal carcinoma.

## Introduction

Esophagus cancer (EC) is one of the most aggressive malignant tumors and is a highly prevalent malignancy worldwide. It ranks first for mortality of digestive system tumors in the United States [1]. The five-year survival rate for EC patients is only 20%, which is slightly higher than the 10% survival rate for patients with pancreatic cancer [1]. EC is divided into two categories: esophagus adenocarcinoma (EADC) and esophagus squamous cell carcinoma (ESCC). EADC accounts for the majority of oesophageal cancer patients in high-income countries. However, more than 90% of oesophageal cancer cases in China are ESCC [2]. The main risk factors for ESCC include smoking, alcohol consumption, nutritional deficiencies, and gene mutations [ 3‒ 5] .

Currently, surgery remains the most effective therapeutic method for patients with EC without metastasis. Unfortunately, most patients are diagnosed at an advanced stage of the disease [ 3, 6] . Metastatic EC is commonly treated with chemotherapy and radiation therapy, which can cause severe side effects [ 7, 8] . However, natural compounds can be a viable alternative for treatment due to their high specificity, low cost, and few side effects.

The antioxidant and antitumor properties of natural compounds found in vegetables, fruits, and herbs are currently being extensively studied [ 9‒ 11] . Previous studies have shown that natural compounds can inhibit the growth of cancer cells and reduce cancer relapse and metastasis in patients with EC [ 12, 13] . Magnolol, also known as 5,5′-diallyl-2,2′-dihydroxybiphenyl, is extracted from *Magnolia officinalis*, a famous traditional Chinese medicine. At low concentrations, it has been found to possess antitumor properties in various cancers, including human hepatocellular carcinoma cells, esophageal cancer cells, and lung squamous carcinoma cells [ 3, 14, 15] .

Autophagy is a conserved metabolic pathway in eukaryotes that plays a crucial role in carcinogenesis and cancer therapy [ 16, 17] . It is widely accepted that autophagy exerts an inhibitory effect during the early stages of tumorigenesis [18]. Autophagy can limit cytoplasmic damage, genomic instability, and inflammation, thereby inhibiting the initiation of tumorigenesis [16]. Loss of certain autophagy-related genes can lead to cancer. For example, *Beclin1* is monoallelically deleted in half of breast, ovarian, and prostate cancers [ 19, 20] . Mutations in the *ATG2B*, *ATG5*, *ATG9B*, and *ATG12* genes are frequently reported in colorectal and gastric cancers [21].

As previously reported, magnolol has been shown to induce autophagy in the non-small cell lung cancer cell line H460 [22]. However, its effect on autophagy in esophageal cancer has not been investigated. This study revealed that magnolol activates autophagy in esophageal cancer cells, and the underlying mechanism was subsequently explored.

## Materials and Methods

### Plasmid construction

The *HACE1* sgRNA was synthesized as oligos (Biosune, Shanghai, China), annealed and ligated into the pX330 vector using T4 DNA ligase (NEB, Ipswich, USA), which was subsequently digested with *Bsm*BI (Thermo Scientific, Waltham, USA); the specific sequences of the sgRNA are shown in Table 1. The promoter region of the human *HACE1* gene, from –1000 to –1, was amplified using the primers listed in Table 2. The amplified region was then ligated into the pGL3-basic vector, which was digested with *Nhe*I and *Xho*I (NEB) and named pGL3-HACE1. The pLKO.1 vectors containing OPTN shRNAs, pGEX4T-1-OPTN/p62 and pET22b-HACE1 were purchased from Cell Researcher Biotech Co., Ltd. (Shanghai, China), and the specific sequences of the shRNAs used are listed in Table 3.

**Table:** **Table 1** Sequences of the sgRNA for *HACE1*

sgRNA	Sequence (5′→3′)
sg HACE1-sense	CACCGGCTGCGCCGCGCGCGCACCG
sg HACE1-antisense	CCGGTGCGCGCGCGGCGCAGCCAAA

**Table:** **Table 2** Sequences of the primers for human *HACE1* gene promoter amplification

Primer	Sequence (5′→3′)
HACE1-P-F	CTATATCCTGAATGGGCAGGTGAGAC
HACE1-P-R	CCGGGGAACTGTAGTTTCCAGCTGG

**Table:** **Table 3** Sequences of the shRNAs targeting *OPTN*

shRNA	Target site sequence (5′→3′)
Scramble	GCGCGATAGCGCTAATAATTT
shOPTN-1	GCCTGTTGTTTGAGATGCAAA
shOPTN-2	GCTTTGCCTAAGGGAAGGAAA

### Cell culture, transfection and reagents

The human EC cell lines KYSE-150 and Eca-109 obtained from the Cell Bank of the Chinese Academy of Sciences (Shanghai, China) were cultured in RPMI-1640 medium (Gibco, Carlsbad, USA) supplemented with 10% fetal bovine serum (FBS; Gibco), 100 mg/mL streptomycin and 100 U/mL penicillin (Gibco) in a humidified incubator with 5% CO _2_ at 37°C. The plasmids were transfected into the cells using Lipofectamine 2000 (Life Technologies, Carlsbad, USA) according to the manufacturer’s instructions. Stably-transfected cells were established using puromycin selection (2 μg/mL; Beyotime, Shanghai, China) for at least 7 days. Stock solutions of magnolol (Sigma-Aldrich, St Louis, USA) and cycloheximide (CHX; Selleck, Houston, USA) were dissolved in dimethyl sulfoxide (DMSO) at 100 mM and stored at ‒80°C prior to use.

### Immunoprecipitation (IP) and immunoblotting (IB)

For the immunoprecipitation assay, KYSE-150 cells were lysed in RIPA buffer [50 mM Tris-HCl, 150 mM NaCl, 5 mM EDTA, 0.1% sodium dodecyl sulfate (SDS), and 1% NP-40, pH 7.6] supplemented with a protease inhibitor cocktail (1:100; Selleck). The cell lysates were then incubated overnight at 4°C with an anti-OPTN antibody (1:200; 10837-1-AP; Proteintech, Chicago, USA) and Protein G magnetic beads (L-1002; Biolinkedin, Shanghai, China) and washed three times with RIPA buffer. The immunoprecipitates were enriched by centrifugation and denatured at 100°C for 15 min in 2× SDS-PAGE protein loading buffer. The immunoprecipitates, inputs and other cell lysates were subjected to SDS-PAGE, and transferred to 0.22-μm PVDF membranes (Millipore, Billerica, USA). The membranes were incubated with the following specific antibodies: anti-LC3 (1:800; 14600-1-AP; Proteintech), anti-GAPDH (1:8000, 60004-1-Ig; Proteintech), anti-caspase-3 (1:1000; 82202-1-RR; Proteintech), anti-HACE1 (1:1000; 24104-1-AP; Proteintech) and anti-ubiquitin (1:1000; sc-47721; Santa Cruz Biotech, Santa Cruz, USA). Then, the membranes were incubated with the corresponding secondary antibodies: horseradish peroxidase-conjugated goat anti-mouse IgG (1:8000; SA00001-1; Proteintech) or goat anti-rabbit IgG (1:8000; SA00001-2; Proteintech). The signals were detected using a Tanon 5200 Imaging System (Tanon, Beijing, China).

### Cell proliferation assay

Two thousand human esophageal carcinoma cells were seeded into a 96-well plate. The 0-h time point was defined as 6 h after seeding. At different time points (0, 24, 48 and 72 h), the cells were incubated with CCK-8 solution (C0037; Beyotime) for 2.5 h at 37°C. The absorbance was detected at a wavelength of 450 nm using a microplate reader (Bio-Rad, Hercules, USA). The cell proliferation assays were conducted in 6 replicates and repeated 3 times.

### Colony formation assay

One thousand human esophageal carcinoma cells were seeded into a 6-well plate. After seven days, the cells were fixed with 4% paraformaldehyde (Sigma-Aldrich) at room temperature for 15 min and then stained with 0.1% crystal violet (C0121; Beyotime) for 30 min. Images were captured using an iPhone 11 (Cupertino, USA), and the number of colonies was quantified.

### Fluorescence microscopy analysis

KYSE-150 and Eca-109 cells were transfected with GFP-LC3 and treated without or with magnolol (30 and 60 μM) for 48 h. The cells were then fixed with 4% paraformaldehyde for 15 min, and the cell nuclei were counterstained with 4,6-diamidino-2-phenylindole (DAPI). Fluorescence was detected using a BX51 microscope (Olympus, Tokyo, Japan). The amount of GFP-LC3 puncta formed was quantified by counting and calculating the number of GFP-LC3 puncta in 10 cells assessed from 10 fields.

### Recombinant protein purification

Glutathione S-transferase (GST)- and hexahistidine (His6)-tagged proteins were purified from the BL21 *E*. *coli* system as described previously [23]. Briefly, after induction with isopropyl-β-d-mercapto-galactopyranoside (Sigma-Aldrich), the cells transfected with protein-encoding plasmids were centrifuged, lysed in PBS buffer (137 mM NaCl, 2.7 mM KCl, 8 mM Na _2_HPO _4_ and 2 mM KH _2_PO _4_, pH 7.6), incubated with glutathione or Ni ^2+^ affinity gels, and eluted with 20 mM reduced L-glutathione solution (pH 8.0) or 300 mM imidazole (pH 8.0). The eluate was then dialyzed against PBS buffer containing 20% glycerol at 4°C overnight before being stored at ‒80°C.

### GST pull-down assay

Purified GST-tagged protein (20 μg), His6-tagged protein (20 μg) and 50 μL of Glutathione Sepharose 4B (Sangon Biotech, Shanghai, China) were incubated at 4°C overnight in 1 mL of GST pull-down buffer [20 mM Tris-HCl, 5 mM MgCl _2_, 100 mM NaCl, 1 mM EDTA, 1% NP-40 and fresh 1 mM dithiothreitol (DTT), pH 7.4] supplemented with 10 mg/L fresh BSA. The samples were pelleted and washed five times with GST pull-down buffer. The immunoprecipitates were then denatured in 50 μL of 2× SDS protein loading buffer at 100°C for 10 min, and then subjected to immunoblotting analysis.

### Data analysis using the GEPIA2 public database

Gene Expression Profiling Interactive Analysis 2 (GEPIA2) ( http://gepia2.cancer-pku.cn) is a tool for gene expression analysis of sequencing data from The Cancer Genome Atlas and the Tissue Genotype Expression database [24]. In this study, GEPIA2 was used to evaluate the mRNA expression of HACE1 and OPTN in human ESCA, and *P* values were calculated using Student’s *t* test; an absolute log _2_(fold change)≥0.5 and *P*<0.05 were considered to indicate a significant difference. Associations between the mRNA expression of *HACE1* or *OPTN* and the pathological stage of ESCA were also assessed using GEPIA2. In addition, GEPIA2 was used for prognostic value analysis by calculating the overall survival (OS) and disease-free survival (DFS) rates, and survival plots were generated directly from GEPIA2 using the log-rank test as the only analysis option.

### Tumor xenograft assay

Four-week-old athymic female nude mice were purchased from Shanghai SLAC Laboratory Animal Co., Ltd (Shanghai, China) and housed for one week before tumor cell injection. All animal studies followed the instructions of the Animal Care and Use Committee of Shanghai Changzheng Hospital, Navy Military Medical University (No. 2022SL011). Tumor cells (Eca-109 and KYSE-150) were subcutaneously injected (1×10 ^7^ cells/mouse) into ten mice per group. The mice bearing tumors were divided into two groups as follows: the control group ( *n*=5) and the magnolol group ( *n*=5). Magnolol treatment was started three days after the inoculation of esophageal carcinoma cells in nude mice. The mice in the magnolol group were injected intraperitoneally with magnolol (30 mg/kg) every other day.

The tumor volumes were measured using a Vernier calliper every three days, and the tumor volume was calculated using the following formula: volume (V)=1/2×(length×width ^2^). On day 21, the mice were sacrificed, and the weights of tumors were measured. The tumors were then lysed in RIPA buffer and subjected to immunoblotting analysis.

### Generation of *HACE1*-knockout (KO) cell lines

*HACE1-*knockout cells were generated using the CRISPR-CAS9-based method as previously described [25]. Briefly, KYSE-150 and Eca-109 cells were transfected with sgRNA (pX330-HACE1-sgRNA) and selected using puromycin (2 μg/mL). Monoclonal cells were then picked, cultured and analyzed by immunoblotting analysis. Genetic ablation of *HACE1* was confirmed by Sanger sequencing.

### Reverse transcription quantitative PCR (RT-qPCR)

Total RNA was extracted from human esophageal carcinoma cells using a total RNA kit (Tiangen). Complementary DNA (cDNA) was synthesized using ReverTra Ace RT Master Mix (Toyobo, Tokyo, Japan). A quantitative PCR (qPCR) assay was performed to assess the relative abundances of *HACE1* and *GAPDH* mRNAs using the specific primers listed in Table 4, and the samples were stained with SYBR Green (Tiangen) on a CFX96 real-time PCR system (Bio-Rad). The relative abundance of *HACE1* mRNA was normalized to that of *GAPDH* using the 2 ^‒ΔΔCt^ method. All data were obtained from three independent experiments.

**Table:** **Table 4** Sequences of the primers used in RT-qPCR

Target gene	Forward primer (5′→3′)	Reverse primer (5′→3′)
*GAPDH*	AGTCAACGGATTTGGTCGTATT	TTTGCCATGGGTGGAATCATAT
*HACE1*	TTGCCCGAGGATAATGAAACTGC	CATTCCACCGATCCACAATTTGCT

### Luciferase reporter assay

KYSE-150 cells were seeded at a density of 2×10 ^5^ cells/well in 12-well plates. The cells were then transiently transfected with pGL3-basic or pGL3-HACE1 along with pRL-TK. After 24 h, the transfected cells were either treated with magnolol or left untreated and cultivated for an additional 24 h. The cells were then harvested and lysed with 5× passive buffer and subjected to dual-luciferase reporter assay using the Promega dual-luciferase reporter assay system (Promega, Madison, USA) according to the manufacturer’s instructions.

### Statistical analysis

The data generated in this study are expressed as the mean±SD and were analyzed using GraphPad Prism 5 (GraphPad Software Inc., San Diego, USA). Statistical significance was determined using a two-tailed unpaired Student’s *t* test or one-way ANOVA with Tukey’s *post hoc* test. A *P* value less than 0.05 was considered to indicate a significant difference, while a *P* value less than 0.01 was considered to indicate a very significant difference.

## Results

### Magnolol induces apoptosis and inhibits the proliferation of EC cells

**Figure 1:**
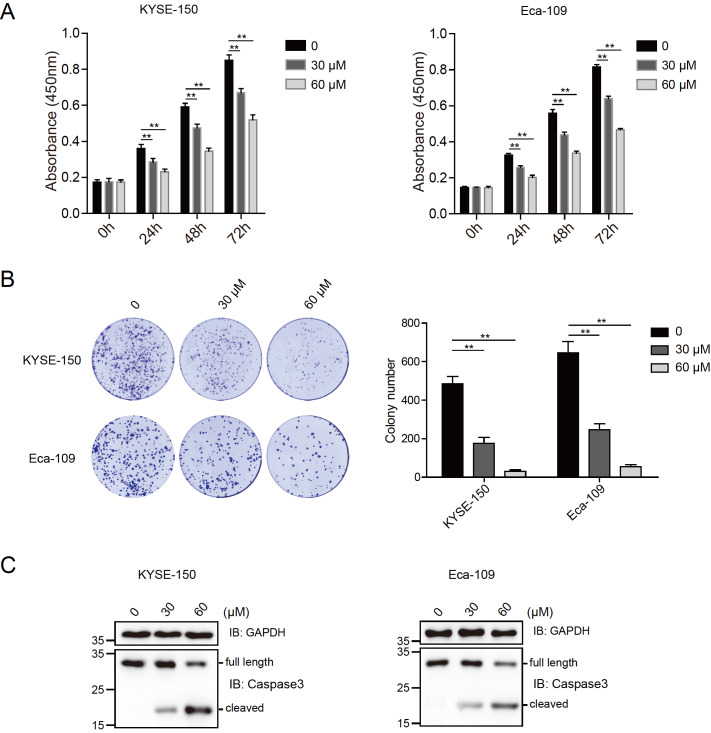
Figure 1 Magnolol induces apoptosis and inhibits the proliferation of EC cells (A) Magnolol inhibited the proliferation of EC cells. The viability of KYSE-150 and Eca-109 cells treated with or without magnolol (30 and 60 μM) for different time intervals (0, 24, 48, and 72 h) was determined by CCK-8 assays. The 0 h time point was defined as 6 h after the cells were seeded into 96-well plates. Data are expressed as the mean±SD and were analyzed using one-way ANOVA with Tukey’s post hoc test. ** P<0.01. (B) Magnolol inhibited the colony formation of EC cells. KYSE-150 and Eca-109 cells were seeded into 6-well plates at 1000 cells/well and cultured for 7 days. The colonies were fixed and stained, and images were acquired using a camera. The number of colonies was counted and calculated. ** P<0.01, n=3. (C) Magnolol activated the apoptosis of EC cells. KYSE-150 and Eca-109 cells were treated with or without magnolol (30 and 60 μM) for 48 h and subjected to immunoblotting analysis using anti-caspase-3 and anti-GAPDH antibodies.

The effect of magnolol on the viability of KYSE-150 and Eca-109 cells was determined by CCK-8 assay at different time points (0, 24, 48, and 72 h) after treatment with different concentrations of magnolol (30 and 60 μM). Significant inhibition of cell growth was observed in magnolol-treated cells compared to control cells ( Figure 1A). In both the KYSE-150 and Eca-109 cell lines, magnolol treatment resulted in reduced colony numbers compared to those in the control groups, as shown by the colony formation assay ( Figure 1B). Additionally, magnolol treatment led to a significant increase in cleaved caspase-3 level in EC cells, indicating the activation of caspase-3-dependent apoptosis ( Figure 1C).

### Magnolol enhances the autophagic activity of EC cells

**Figure 2:**
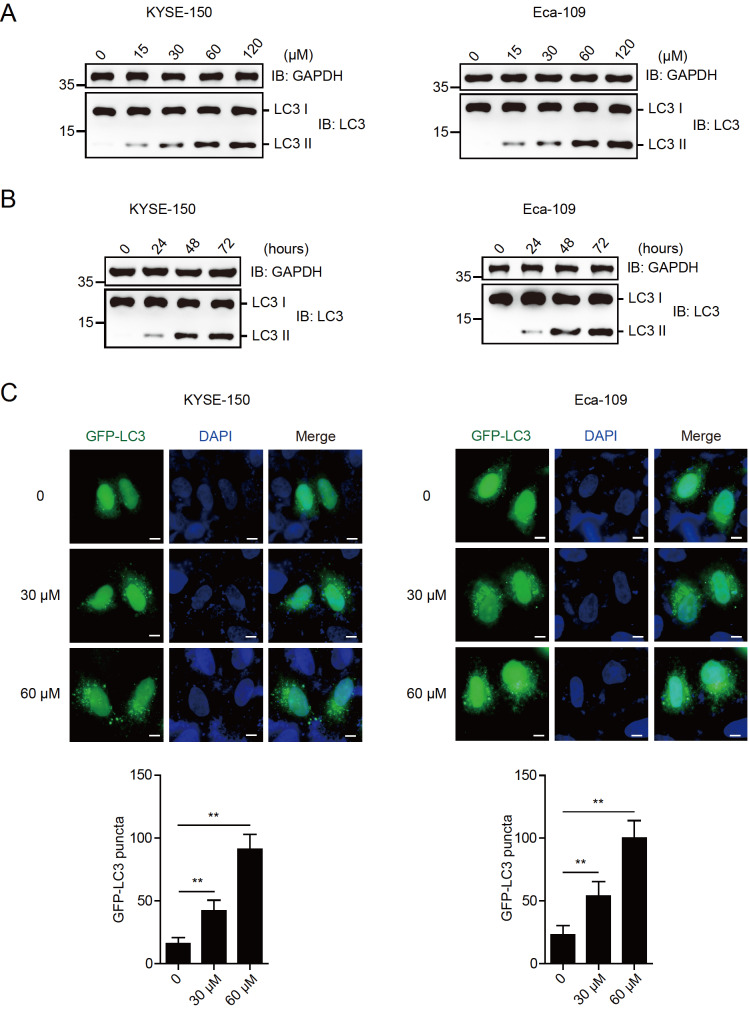
Figure 2 Magnolol enhances the autophagic activity of EC cells (A) Magnolol promoted autophagy in EC cells in a dose-dependent manner. KYSE-150 and Eca-109 cells were treated with different doses of magnolol (0, 15, 30, 60 and 120 μM) for 48 h and subjected to immunoblotting analysis using anti-LC3 and anti-GAPDH antibodies. (B) Magnolol promoted autophagy in EC cells in a time-dependent manner. KYSE-150 and Eca-109 cells were treated with 60 μM magnolol for different time intervals (0, 24, 48, and 72 h) and subjected to immunoblotting analysis using the indicated antibodies. (C) GFP-LC3 puncta formation was enhanced in EC cells treated with magnolol. KYSE-150 and Eca-109 cells were transfected with GFP-LC3, treated without or with magnolol (30 and 60 μM) for 48 h and subjected to fluorescence microscopy analysis. GFP-LC3 puncta were counted, and the number of colonies was calculated. ** P<0.01. Scale bar: 10 μm.

To investigate the effect of magnolol on autophagy in EC, KYSE-150 and Eca-109 cells were treated with different concentrations of magnolol (0, 15, 30, 60 and 120 μM) for 48 h. The level of lipidated LC3 (LC3 II) increased in a magnolol dose-dependent manner ( Figure 2A). A high level of autophagy was observed in both KYSE-150 and Eca-109 cells treated with 60 μM magnolol, which was selected for further studies. KYSE-150 and Eca-109 cells were treated with 60 μM magnolol for different time periods (0, 24, 48, and 72 h). LC3 II level was increased in a time-dependent manner. Treatment of cells with magnolol for 48 h increased autophagy ( Figure 2B). Therefore, this time point was selected for subsequent experiments. A functional autophagy assay was conducted by transfecting KYSE-150 and Eca-109 cells with GFP-LC3. The cells were then treated with magnolol (30 and 60 μM) for 48 h and subjected to fluorescence microscopy with or without treatment. The results showed that magnolol treatment enhanced the puncta formation of GFP-LC3 in esophageal carcinoma cells ( Figure 2C). These data suggest that magnolol effectively activates autophagy in EC cells.

### Magnolol inhibits the proliferation of EC cells in a HACE1-dependent manner

**Figure 3:**
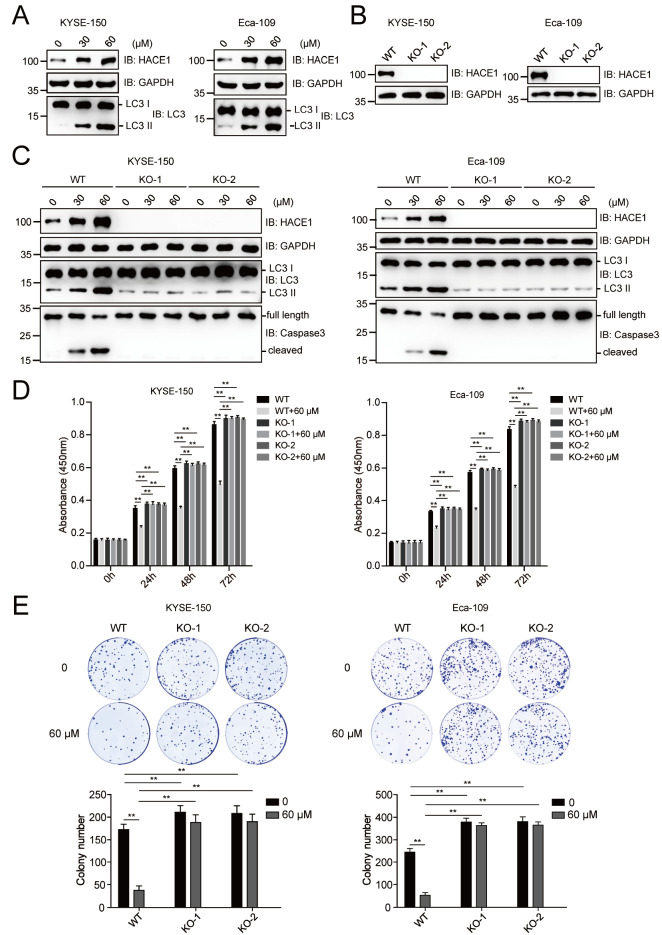
Figure 3 Magnolol inhibits the proliferation of EC cells in a HACE1-dependent manner (A) Magnolol increased the protein level of HACE1. KYSE-150 and Eca-109 cells were treated with or without magnolol (30 and 60 μM) for 48 h and subjected to immunoblotting analysis using anti-HACE1, anti-LC3 and anti-GAPDH antibodies. (B) HACE1 knockout (KO) cell lines were generated using the CRISPR-CAS9 gene editing approach. KYSE-150 and Eca-109 cells were transfected with CRISPR-CAS9-based sgRNA, and monoclonal sequences were picked and detected by immunoblotting analysis. WT, wild type; KO, knockout. (C) Magnolol lost its ability to promote autophagy in HACE1-KO EC cells. KYSE-150 and Eca-109 cells with or without HACE1-KO were treated with magnolol (30 and 60 μM) for 48 h and subjected to immunoblotting analysis using the indicated antibodies. (D) Magnolol inhibited the proliferation of wild-type but not HACE1-KO EC cells. The viability of HACE1-knockout or untreated KYSE-150 and Eca-109 cells treated with or without 60 μM magnolol for different time intervals (0, 24, 48 and 72 h) was determined by the CCK-8 assay. The 0 h time point was defined as 6 h after the cells were seeded into 96-well plates. ** P<0.01. (E) Magnolol inhibited the colony formation of wild-type but not HACE1-KO EC cells. KYSE-150 and Eca-109 cells with or without HACE1-KO were seeded in 6-well plates and cultured for 7 days. The colonies were fixed and stained, and images were captured with a camera. The number of colonies was counted and calculated. ** P<0.01, n=3.

A previous study reported that the ubiquitination of the autophagy receptor OPTN (optineurin) by HACE1 activates selective autophagy for tumor suppression [26]. We then tested the effect of magnolol on the protein expression of HACE1. The results showed that magnolol promoted LC3 II level, accompanied by increased HACE1 protein level ( Figure 3A). We generated the *HACE1*-knockout (KO) cell lines KYSE-150 and Eca-109 using the CRISPR-CAS9 sgRNA-based method ( Figure 3B). The ablation of *HACE1* almost completely eliminated the magnolol-induced increase in autophagy and caspase-3-dependent apoptosis ( Figure 3C). Magnolol inhibited the proliferation of wild-type KYSE-150 and Eca-109 cells. However, this effect was lost in *HACE1*-KO cells, as shown by the CCK-8 assay ( Figure 3D). Consistently, *HACE1* ablation weakened the ability of magnolol to inhibit EC cell clonogenesis ( Figure 3E).

### Magnolol promotes autophagy in EC cells through the HACE1-OPTN axis

**Figure 4:**
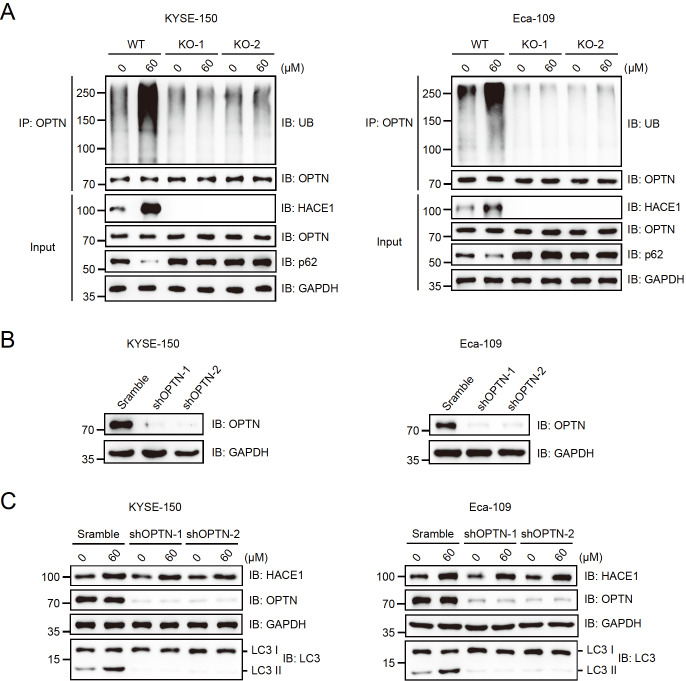
Figure 4 Magnolol promotes the autophagy of EC cells through the HACE1-OPTN axis (A) Magnolol promoted the ubiquitination of the autophagy receptor OPTN in wild-type but not HACE1-KO EC cells. KYSE-150 and Eca-109 cells with or without HACE1-KO were treated with or without 60 μM magnolol for 48 h, and the lysates were immunoprecipitated with an anti-OPTN antibody before immunoblotting with the indicated antibodies. (B) The knockdown efficiency of shRNAs targeting OPTN was tested by immunoblotting analysis. shRNAs targeting OPTN were transfected into KYSE-150 and Eca-109 cells, and puromycin selection was used to establish stable expression cell lines. Scramble, negative control. (C) Magnolol lost the ability to promote autophagy in the OPTN-KO EC cells. KYSE-150 and Eca-109 cells with or without OPTN-KO were treated with or without 60 μM magnolol for 48 h and subjected to immunoblotting analysis using the indicated antibodies.

Magnolol increased the protein level of HACE1, promoting OPTN ubiquitination in wild-type (WT) KYSE-150 and Eca-109 cells. The autophagy receptor p62 was decreased in WT cells but not in *HACE1*-KO cells under magnolol treatment ( Figure 4A). GST pull-down assay demonstrated that HACE1 directly interacts with OPTN but not with p62 ( Supplementary Figure S1A). This finding suggested that magnolol regulates autophagy through HACE1-mediated ubiquitination and degradation of p62.

Two shRNAs were constructed to target OPTN and were tested in KYSE-150 and Eca-109 cells ( Figure 4B). These shRNAs exhibited high knockout efficiency and were used to establish stable cell lines for further research. As demonstrated in Figure 4C, magnolol was unable to promote autophagy in *OPTN*-knockout esophageal carcinoma cells. These results suggest that magnolol primarily enhances the autophagy activity of esophageal carcinoma cells through the HACE1-OPTN axis.

The mRNA expression levels of *HACE1* and *OPTN* in human ESCA were analyzed using the GEPIA2 database, and no significant differences were found compared to those in the corresponding normal tissues ( Supplementary Figure S1B). We further analyzed the associations between HACE1 or OPTN and the pathological stage of ESCA using GEPIA2. No significant differences were found ( Supplementary Figure S1C). Furthermore, patients with high HACE1 or OPTN expression did not exhibit any significant differences in overall or disease-free survival compared to patients with low HACE1 or OPTN expression ( Supplementary Figure S1D,E). It is important to note that the GEPIA2 database only contains information on mRNA expressions and not on protein expressions.

### Magnolol suppresses the tumorigenicity of human EC cells in nude mice by elevating HACE1 and LC3 II protein levels

**Figure 5:**
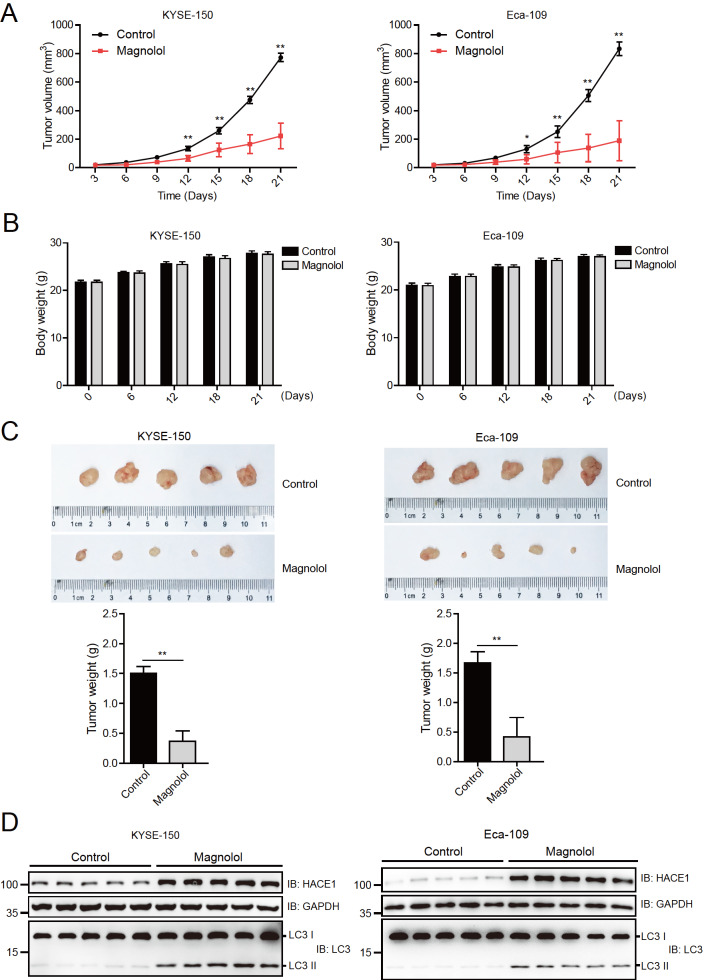
Figure 5 Magnolol suppresses the tumorigenicity of human EC cells in nude mice by elevating HACE1 and LC3 II protein levels (A) The tumor volume in nude mice was decreased by magnolol. KYSE-150 and Eca-109 cells were injected into nude mice, which were treated with magnolol (30 mg/kg) every other day, and the volume of the tumors was measured with a Vernier calliper every 3 days. * P<0.05, ** P<0.01, n=5. (B) Magnolol had no obvious effect on the body weight of the nude mice. The body weights of the nude mice were measured on different days after tumor inoculation. Data are expressed as the mean±SD and were analyzed using a two-tailed unpaired Student’s t test. No significant differences were found. (C) Images showing the xenograft tumors of nude mice injected with KYSE-150 and Eca-109 cells at 21 days. The weights of the tumors were measured and calculated, and data are expressed as the mean±SD and were analyzed using two-tailed unpaired t tests. ** P<0.01, n=5. (D) Magnolol increased the protein levels of HACE1 and LC3 II in xenograft tumors. The protein levels of HACE1 and LC3 were detected by immunoblotting analysis in xenograft tumors, from Figure 5C.

To investigate the impact of magnolol on human EC cells, a tumorigenicity model was established in nude mice. KYSE-150 and Eca-109 cells were injected into nude mice, which were then treated with magnolol (30 mg/kg) every other day. As shown in Figure 5A, the tumors in the control groups grew rapidly compared to those in the magnolol-treated groups. The body weights of the nude mice were measured on different days after tumor inoculation, and no significant differences were observed ( Figure 5B). At 21 days after injection, the tumors in the magnolol-treated groups were significantly lighter than those in the control groups ( Figure 5C). However, it should be noted that the homogeneity of tumor size in the magnolol group of Eca-109 cells was not good. Immunoblotting analysis of the lysates of these tumors revealed higher protein levels of HACE1 and LC3 II in the magnolol-treated groups than in the control groups ( Figure 5D).

### Magnolol enhances the transcription of *HACE1*

**Figure 6:**
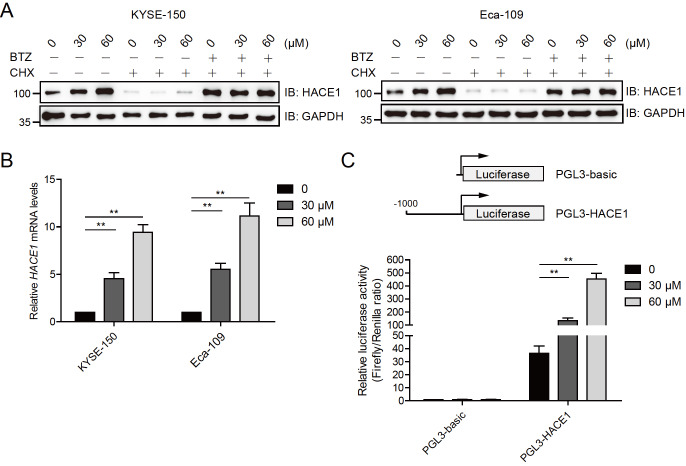
Figure 6 Magnolol enhances the transcription of HACE1 (A) Magnolol did not promote HACE1 expression in esophageal carcinoma cells treated with protein translation inhibitors. KYSE-150 and Eca-109 cells were pretreated with CHX for 2 h, followed by simultaneous treatment with or without CHX, BTZ, and magnolol (30 and 60 μM) for 24 h. The protein levels of HACE1 and GAPDH were determined by immunoblotting analysis. CHX, protein synthesis inhibitor cycloheximide; BTZ, proteasome inhibitor bortezomib. (B) Magnolol promoted the expression of the HACE1 gene. KYSE-150 and Eca-109 cells were treated with or without magnolol (30 and 60 μM) for 48 h and subjected to RT-qPCR analysis. ** P<0.01, n=3. (C) Magnolol promoted the luciferase activity of the HACE1 gene promoter. The ‒1000 to ‒1 region of the HACE1 gene promoter was amplified and inserted into the pGL3-basic vector, which was named pGL3-HACE1. KYSE-150 cells were transfected with pGL3-basic or pGL3-HACE1 for 24 h and then treated with or without magnolol (30 and 60 μM) for 48 h before being subjected to luciferase activity assay. ** P<0.01, n=3.

The data presented above indicated that magnolol promotes the expression of HACE1. The mechanism by which magnolol promotes HACE1 expression was preliminarily explored by pretreating KYSE-150 and Eca-109 cells with the protein synthesis inhibitor cycloheximide (CHX), followed by simultaneous treatment with or without CHX, bortezomib (BTZ), and magnolol. Figure 6A shows that CHX effectively blocked the effect of magnolol, indicating that magnolol does not affect protein translation. Additionally, quantitative PCR (qPCR) analysis revealed that magnolol promoted the expression of the *HACE1* gene in both KYSE-150 and Eca-109 cells ( Figure 6B). The promoter region (‒1000 to ‒1) of the *HACE1* gene was cloned and inserted into the pGL3-basic vector, and luciferase activity was measured. Magnolol significantly increased the luciferase activity of the *HACE1* gene promoter (pGL3-HACE1) but had little effect on the pGL3-basic vector ( Figure 6C). These results suggested that magnolol promotes HACE1 expression at the transcriptional level.

## Discussion

Accumulating evidence suggests that natural compounds, including small and large molecules extracted and isolated from natural products, play a crucial role in cancer treatment. They can inhibit cancer cell proliferation and induce apoptosis, as well as prevent cancer cell metastasis and angiogenesis [ 18, 27, 28] . For instance, dihydroartemisinin (DHA) has been shown to have anticancer effects on esophageal cancer cells through autophagy-dependent cell cycle arrest [29]. Like DHA, magnolol inhibits the proliferation of esophageal carcinoma cells by inducing autophagy. Additionally, magnolol activated autophagy at low concentrations ( Figures 1 and 2). A previous study reported that high concentrations (more than 40 mM) of magnolol induced autophagy in the H460 non-small cell lung cancer cell line [22]. This suggests that magnolol may have diverse effects on different tumors, but it can indeed induce autophagy.

Our previous study revealed that magnolol can induce apoptosis in esophageal cancer [3]. In this study, we found that magnolol mainly inhibits tumor growth by inducing apoptosis. However, further research is needed to explore the underlying mechanism of magnolol, including its effects on autophagy and apoptosis.

As previously reported, HACE1 functions as a tumor suppressor by ubiquitinating OPTN and activating selective autophagy [26]. HACE1 is frequently lost or downregulated in many tumors, including lung and liver cancers [30]. In our study, magnolol was found to promote the expression of HACE1 in both esophageal carcinoma cells and xenograft tumors ( Figures 3A and 5D). The ablation of *HACE1* and *OPTN* abolished the ability of magnolol to activate autophagy ( Figures 3C and 4C). The ubiquitin‒proteasome pathway is a selective protein degradation pathway that plays a crucial role in cell proliferation, differentiation, and metabolism [ 31, 32] . Dysfunctions in ubiquitination have been linked to various cancers, including esophageal cancer [33]. Our study revealed that magnolol promoted the ubiquitination of OPTN, accompanied by decreased p62 level ( Figure 4A). Our data suggest that magnolol mainly induces autophagy by activating the HACE1-OPTN axis. In our study, *HACE1* knockout almost completely reversed the antiproliferative effect of magnolol, as detected by CCK-8 assays ( Figure 3D). However, the colony formation assay showed only a partial rescue effect ( Figure 3E). This difference could be due to the length of the observation period, as longer observation periods tend to reveal more significant differences.

Magnolol promotes the expression of HACE1 primarily at the transcriptional level, as demonstrated by qPCR and luciferase activity ( Figure 6B,C). The underlying molecular mechanism is still unknown, but one possible explanation is that magnolol increases the expression of MBD3. MBD3 is capable of binding to the *HACE1* gene promoter and promoting demethylation [34]. Previous reports suggest that MBD3 interacts with TET2, promoting its demethylase activity and resulting in increased expression of various genes, including *GNRH1* [35]. However, further experiments are required to verify this assumption.

There are still several unanswered questions regarding magnolol. How magnolol enters tumor cells and whether it acts directly or through its metabolites inside the cells remain unclear. Numerous magnolol analogues, including propyl magnolol, isopropyl magnolol, butyl magnolol, and isobutyl magnolol, have been reported [ 36, 37] . It is uncertain whether these analogues also promote autophagy by enhancing the expression of *HACE1*. These issues require further investigation and will be the focus of our future research.

## Supporting information

23602supplementary_Figure_1

## Supplementary Data

Supplementary data is available at *Acta Biochimica et Biophysica Sinica* online.

## References

[1] Siegel RL, Miller KD, Fuchs HE, Jemal A (2021). Cancer statistics, 2021. CA Cancer J Clin.

[2] Abnet CC, Arnold M, Wei WQ (2018). Epidemiology of esophageal squamous cell carcinoma. Gastroenterology.

[3] Chen Y, Huang K, Ding X, Tang H, Xu Z (2019). Magnolol inhibits growth and induces apoptosis in esophagus cancer KYSE-150 cell lines via the MAP kinase pathway. J Thorac Dis.

[4] Xu A, Sun M, Li Z, Chu Y, Fang K, Zhang Y, Lian J (2023). ELF4 contributes to esophageal squamous cell carcinoma growth and metastasis by augmenting cancer stemness via FUT9. Acta Biochim Biophys Sin.

[5] Liang F, Luo Q, Han H, Zhang J, Yang Y, Chen J (2023). Long noncoding RNA LINC01088 inhibits esophageal squamous cell carcinoma progression by targeting the NPM1-HDM2-p53 axis. Acta Biochim Biophys Sin.

[6] Niu C, Liu Y, Wang J, Liu Y, Zhang S, Zhang Y, Zhang L (2021). Risk factors for esophageal squamous cell carcinoma and its histological precursor lesions in China: a multicenter cross-sectional study. BMC Cancer.

[7] Santeufemia DA, Tumolo S, De Paoli A, Lo Re G, Boz G, Miolo GM, Baresic T,
*et al*. Chemo/tomotherapy stereotactic body radiation therapy (chemo/SBRT) for the salvage treatment of esophageal carcinoma following trimodality therapy: a case report.
*
Tumori
* 2012, 98: 143e–145e. https://doi.org/10.1700/1190.13217.

[8] Rochigneux P, Tyran M, Autret A, Lopez Almeida L, Guiramand J, Ferre M, Chanez B (2022). Impact of fiducial markers placement on the delineation of target volumes in radiation therapy for oesophageal cancer: FIDUCOR study. Front Oncol.

[9] Li S, Shen XY, Ouyang T, Qu Y, Luo T, Wang HQ (2017). Synergistic anticancer effect of combined crocetin and cisplatin on KYSE-150 cells via p53/p21 pathway. Cancer Cell Int.

[10] Lin SR, Fu YS, Tsai MJ, Cheng H, Weng CF (2017). Natural compounds from herbs that can potentially execute as autophagy inducers for cancer therapy. Int J Mol Sci.

[11] Hyuga S, Hyuga M, Oshima N, Maruyama T, Kamakura H, Yamashita T, Yoshimura M (2016). Ephedrine alkaloids-free ephedra herb extract: a safer alternative to ephedra with comparable analgesic, anticancer, and anti-influenza activities. J Nat Med.

[12] Wu T, Yang X, Zeng X, Eslick GD (2009). Traditional chinese medicinal herbs in the treatment of patients with esophageal cancer: a systematic review. Gastroenterol Clin N Am.

[13] Liu J, Yue J (2014). Preliminary study on the mechanism of oridonin-induced apoptosis in human squamous cell oesophageal carcinoma cell line EC9706. J Int Med Res.

[14] Yang SE, Hsieh MT, Tsai TH, Hsu SL (2003). Effector mechanism of magnolol-induced apoptosis in human lung squamous carcinoma CH27 cells. Br J Pharmacol.

[15] Wang YD, Sun XJ, Yang WJ, Li J, Yin JJ (2018). Magnolol exerts anticancer activity in hepatocellular carcinoma cells through regulating endoplasmic reticulum stress-mediated apoptotic signaling. Onco Targets Ther.

[16] Liu EY, Ryan KM (2012). Autophagy and cancer-issues we need to digest. J Cell Sci.

[17] Wang CC, Peng H, Wang Z, Yang J, Hu RG, Li CY, Geng WJ (2022). TRIM72-mediated degradation of the short form of p62/SQSTM1 rheostatically controls selective autophagy in human cells. Military Med Res.

[18] Xie Q, Chen Y, Tan H, Liu B, Zheng LL, Mu Y (2021). Targeting autophagy with natural compounds in cancer: a renewed perspective from molecular mechanisms to targeted therapy. Front Pharmacol.

[19] Aita VM, Liang XH, Murty VVVS, Pincus DL, Yu W, Cayanis E, Kalachikov S (1999). Cloning and genomic organization of beclin 1, a candidate tumor suppressor gene on chromosome 17q21. Genomics.

[20] Liang XH, Jackson S, Seaman M, Brown K, Kempkes B, Hibshoosh H, Levine B (1999). Induction of autophagy and inhibition of tumorigenesis by beclin 1. Nature.

[21] Kang MR, Kim MS, Oh JE, Kim YR, Song SY, Kim SS, Ahn CH (2009). Frameshift mutations of autophagy-related genes ATG2B, ATG5, ATG9B and ATG12 in gastric and colorectal cancers with microsatellite instability. J Pathol.

[22] Li H, Yi X, Gao J, Ying X, Guan H, Li J (2007). Magnolol-lnduced H460 cells deathvia autophagy but not apoptosis. Arch Pharm Res.

[23] Yang Y, Luo Y, Yang C, Hu R, Qin X, Li C (2023). TRIM25-mediated ubiquitination of G3BP1 regulates the proliferation and migration of human neuroblastoma cells. Biochim Biophys Acta Gene Regul Mech.

[24] Tang Z, Li C, Kang B, Gao G, Li C, Zhang Z (2017). GEPIA: a web server for cancer and normal gene expression profiling and interactive analyses. Nucleic Acids Res.

[25] Xu X, Li C, Gao X, Xia K, Guo H, Li Y, Hao Z (2018). Excessive UBE3A dosage impairs retinoic acid signaling and synaptic plasticity in autism spectrum disorders. Cell Res.

[26] Liu Z, Chen P, Gao H, Gu Y, Yang J, Peng H, Xu X (2014). Ubiquitylation of autophagy receptor optineurin by hace1 activates selective autophagy for tumor suppression. Cancer Cell.

[27] Liu Y, Hua W, Li Y, Xian X, Zhao Z, Liu C, Zou J (2020). Berberine suppresses colon cancer cell proliferation by inhibiting the SCAP/SREBP-1 signaling pathway-mediated lipogenesis. Biochem Pharmacol.

[28] Kang DY, Sp N, Lee JM, Jang KJ (2021). Antitumor effects of ursolic acid through mediating the inhibition of stat3/pd-l1 signaling in non-small cell lung cancer cells. Biomedicines.

[29] Ma Q, Liao H, Xu L, Li Q, Zou J, Sun R, Xiao D (2020). Autophagy-dependent cell cycle arrest in esophageal cancer cells exposed to dihydroartemisinin. Chin Med.

[30] Yu Z, Li Y, Han T, Liu Z (2019). Demethylation of the HACE1 gene promoter inhibits the proliferation of human liver cancer cells. Oncol Lett.

[31] Mansour MA (2018). Ubiquitination: friend and foe in cancer. Int J Biochem Cell Biol.

[32] Sheng X, Xia Z, Yang H, Hu R (2024). The ubiquitin codes in cellular stress responses. Protein Cell.

[33] Cheng W, Li G, Ye Z, Hu J, Gao L, Jia X, Zhao S (2022). NEDD4L inhibits cell viability, cell cycle progression, and glutamine metabolism in esophageal squamous cell carcinoma via ubiquitination of c-Myc. Acta Biochim Biophys Sin.

[34] Li S, Yang H, Zhao M, Gong L, Wang Y, Lv Z, Quan Y (2020). Demethylation of
*HACE1* gene promoter by propofol promotes autophagy of human A549 cells. Oncol Lett.

[35] Li C, Lu W, Yang L, Li Z, Zhou X, Guo R, Wang J (2020). MKRN3 regulates the epigenetic switch of mammalian puberty via ubiquitination of MBD3. Natl Sci Rev.

[36] Rycek L, Puthenkalam R, Schnürch M, Ernst M, Mihovilovic MD (2015). Metal-assisted synthesis of unsymmetrical magnolol and honokiol analogs and their biological assessment as GABAA receptor ligands. Bioorg Med Chem Lett.

[37] Syu WJ, Shen CC, Lu JJ, Lee GH, Sun CM (2004). Antimicrobial and cytotoxic activities of neolignans from magnolia officinalis. Chem Biodiversity.

