# Corrigendum to: Magnolol promotes the autophagy of esophageal carcinoma cells by upregulating HACE1 gene expression

**DOI:** 10.3724/abbs.2025129

**Published:** 2025-09-25

**Authors:** Kenan Huang, Biao Zhang, Yu Feng, Haitao Ma

**Affiliations:** 1 Department of Thoracic Surgery Dushu Lake Hospital Affiliated to Soochow University Suzhou 215000 China; 2 Department of Thoracic Surgery Shanghai Changzheng Hospital Navy Military Medical University Shanghai 200003 China

*Acta Biochim Biophys Sin* 2024, 56(7): 1044–1054. 

https://doi: 10.3724/abbs.2024044. 

In the originally published version, the corresponding author’s email address was personal. To comply with our institution’s publishing policy, it has been changed from “mahaitao_123@163.com” to the institutional address “mahaitao@suda.edu.cn”. The authors apologize for any confusion it may have caused.

