# Supplementary figures and images for: Magnolol promotes the autophagy of esophageal carcinoma cells by upregulating HACE1 gene expression : Magnolol activates HACE1-mediated autophagy
Source: Acta Biochim Biophys Sin (Shanghai). 2024 Apr 25;56(7):1044–54. doi: 10.3724/abbs.2024044 (PMC11322865; doi:10.3724/abbs.2024044)

A

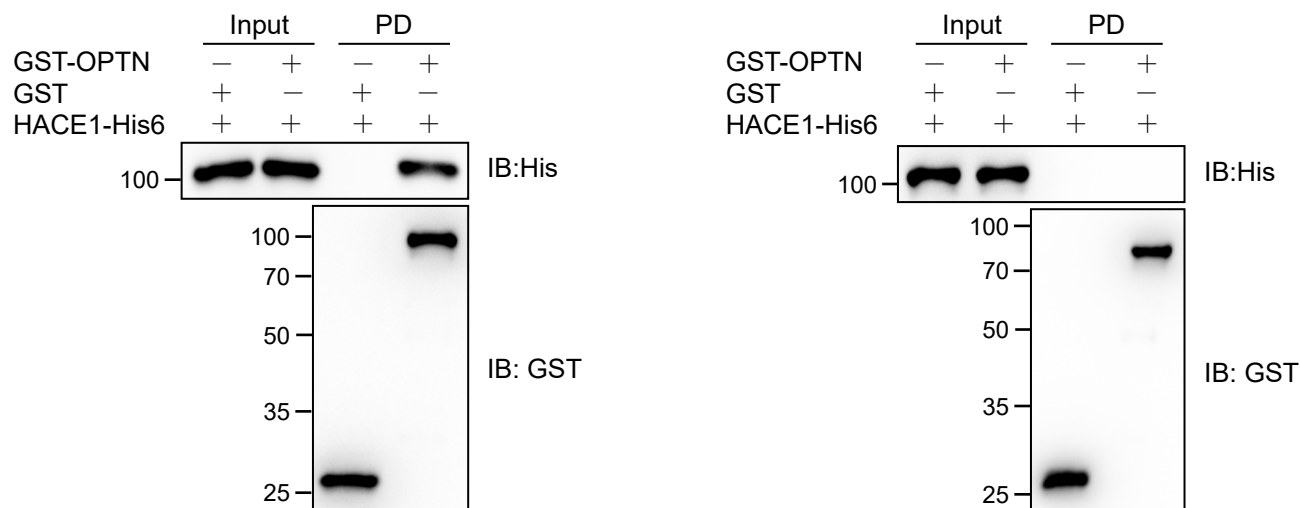

B

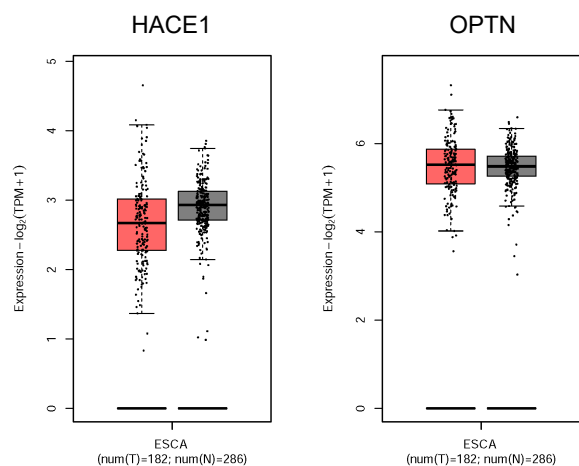

C

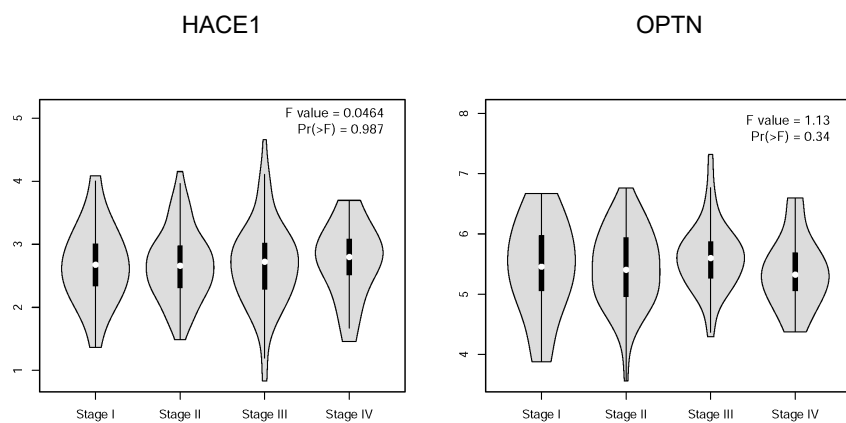

D

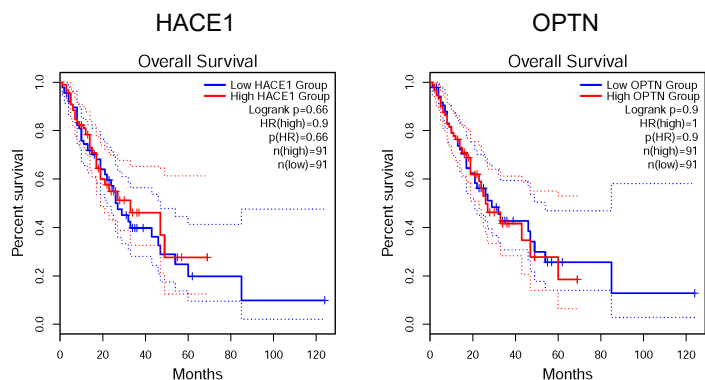

E

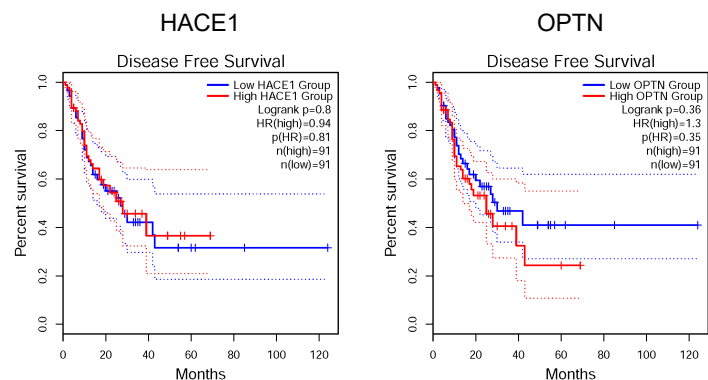

Supplement: 23602supplementary_Figure_1 [file 23602supplementary_Figure_1.pdf]
